# Prime effects in metaphor comprehension: comparing congruent and opposite schematic primes

**DOI:** 10.3389/fpsyg.2024.1355045

**Published:** 2024-11-25

**Authors:** Omid Khatin-Zadeh, Danyal Farsani, Zahra Eskandari, Lin Li, Hassan Banaruee

**Affiliations:** ^1^School of Foreign Languages, University of Electronic Science and Technology of China, Chengdu, China; ^2^Department of Teacher Education, Norwegian University of Science and Technology, Trondheim, Norway; ^3^School of Foreign Languages, Southwestern University of Finance and Economics, Chengdu, China; ^4^Department of Educational Psychology, University of Education Weingarten, Weingarten, Germany

**Keywords:** metaphor comprehension, congruent literal prime, opposite literal prime, congruent gesture prime, opposite gesture prime

## Abstract

This study investigates the role of priming in the process of metaphor comprehension focusing on both literal and gesture-based primes under congruent and opposite conditions. We conducted a two-stage experiment to explore how different priming conditions influence the cognitive processing of metaphors. In stage 1, participants made sensibility judgments on a set of metaphors in congruent literal primes (Group 1), opposite literal primes (Group 2), and no-prime conditions, with Group 3 serving as a baseline. In stage 2, participants performed the same task under congruent gesture-prime (Group 4) and opposite gesture-prime conditions (Group 5), again with Group 3 as the baseline. Sensibility judgments and reaction times were analyzed and compared across all five conditions. Findings of stage 1 reveal that congruent literal primes facilitate process of metaphor comprehension, whereas opposite literal primes delay the process of understanding the subsequent metaphor. Similarly, results of stage 2 show that congruent gesture primes facilitate the process of understanding the subsequent metaphor, while opposite gesture primes delay it. These results align with theories of embodied metaphor comprehension, highlighting the varying influences of primes on metaphor comprehension.

## 1 Introduction

The concept of *image schema* is one of the fundamental construct in cognitive science and related disciplines. Initially discussed by Johnson (1987) and Lakoff (1987), image schemas are defined as recurring cognitive patterns that structure our understanding, including but not limited to concepts such as containment, blockage, counterforce, verticality, and rotation. Early research characterized image schemas as dynamic structures that are formed in our cognitive system as a result of perception, body movement, object manipulation, and the experience of force (Mandler and Pagán Cánovas, 2014). Gibbs and Colston (1995, p. 349) describe image schemas as “analog representations of spatial relations and movements in space.” Gibbs (2006, p. 91) further elaborates that “image schemas are more abstract than ordinary visual mental images and consist of dynamic spatial patterns that underlie the spatial relations and movement found in actual concrete images” (see also, Bolognesi, 2017; Cheng et al., 2024; Kövecses, 2017; Khatin-Zadeh et al., 2023a; Langacker, 2016; Mandler, 2010; Vergaro et al., 2014; Watson et al., 2014). Based on these definitions, an image schema is an abstract, high-level, or general structure that is shared across various concrete phenomena, formed in the mind as a result of repeated processing of those concrete phenomena. In essence, an image schema can be conceived as the abstract representation of multiple concrete phenomena that share an underlying structure. In other words, an image schema is a basic structural similarity between a set of concrete phenomena.

Building on this theoretical foundation, Khatin-Zadeh and Khoshsima (2021) introduced the concept of homo-schematic metaphors, refering to metaphors that share the same underlying schema. For instance, the metaphors *prices are going up*, *Thomas is moving fast to the peak of success*, and *Philip ascended the throne* share the schema *X moves upward*. While these metaphors have distinct meanings and are used in different contexts, they are unified by the schema of upward movement of an object. Similarly, the metaphors *the board is pushing the president to make the decision*, *he is pushing his plans forward*, and *John is pushing his argument* share the schema *X pushes Y forward*. These examples demonstrate how homo-schematic metaphors are grounded in abstract structural similarities that can be the base for creating a large set of homo-schematic metaphors. This notion also aligns with theories of generic metaphors (see Khatin-Zadeh et al., 2022, p. 60) which propose that “there is a deep homogeneity among a set of concretely different metaphors.” Furthermore, not only metaphors but also literal statements can share the same underlying schema. For example, the sentences *mountaineers are moving toward the peak* and *he is moving toward the peak of success* share the schema *X moves upward*. However, the former is a literal expression and has a literal schema, whereas the latter is a metaphorical one and has a metaphor schema. A literal schema directly represents a concrete event or object, whereas a metaphorical schema involves the abstract description of an event or concept through metaphorical mapping.

Gestures can also represent either literal or metaphorical schemas. For example, the literal sentences *Jim climbed up the tree* and *the mountaineers ascended the path up the mountain* can be accompanied by a gesture depicting an upward movement. Similarly, metaphors like *prices are going up* and *he is climbing the ladder of success* may be complemented by the same upward movement gesture. In fact, a gesture that depicts an upward movement can be shared by a range of both literal and metaphorical statements. This study aims to investigate how the process of understanding a metaphor is influenced by preceding literal or gestural primes. In the literal-prime conditions (Stage 1 of our experiment), each metaphor was preceded by a literal statement that either shared the same schema (congruent literal-prime condition) or an opposite schema (opposite literal-prime condition) as the subsequent metaphor. In the gesture-prime conditions (Stage 2 of our experiment), each metaphor was preceded by a gesture that either matched (congruent gesture-prime condition) or contradicted (opposite gesture-prime condition) the schema of the metaphor. The first one was called congruent gesture-prime conditions and the second one was called opposite gesture-prime conditions. The subsequent section reviews previous related priming studies on metaphors and outlines the methodology employed in this study.

## 2 Priming studies on metaphor processing

Priming studies represent a distinct research avenue aimed at elucidating the cognitive processes underlying metaphor comprehension. Generally, prime is a stimulus presented to an individual to observe its influence on an immediate subsequent task. In one of priming studies conducted on metaphor comprehension processes, Khatin-Zadeh and Khoshsima (2021) investigated sensibility judgments across three conditions: no-prime, literal-prime, and metaphor-prime. In the literal-prime condition, participants were asked to judge the sensibility of a metaphor after being exposed to a literal statement displayed on a computer screen. In the metaphor-prime condition, participants evaluated a metaphor after seeing a homo-schematic metaphor on the screen. The researchers found that the metaphor-prime conditions yielded the highest degree of metaphor sensibility and the fastest judgment times. They proposed that this effect resulted from the activation of a shared metaphor schema between the target metaphor and its homo-schematic prime. In a related study examining the role of metaphor schemas in metaphor comprehension, Khatin-Zadeh et al. (2023b) assessed the impact of gestural primes. In this study, three groups of participants made judgments on the sensibility of a set of metaphors under three conditions: congruent gesture-prime, incongruent gesture-prime, and no-prime. In the congruent gesture- prime condition, the gesture represented the schema of the subsequent metaphor; while in the incongruent gesture-prime conditions, the gesture was not congruent with the schema of the subsequent metaphor. The results indicated that congruent gesture primes facilitated faster sensibility judgments and the metaphors were considered to be more sensible underscoring the influence of schematic alignment in metaphor comprehension (see also Khatin-Zadeh, 2023). These findings suggest that verbal and gestural primes can significantly impact the metaphor comprehension processes. While some primes can facilitate metaphor comprehension, others may disrupt this process.

In two priming experiments conducted by Wilson and Gibbs (2007), the impact of real or imagined body movements on the immediate processing of subsequent metaphors was studied. Their findings demonstrated that performing or imagining appropriate body movements facilitated the process of understanding subsequent metaphors. For example, a real pushing action done by the comprehender or imagining a pushing action facilitated the process of understanding the metaphorical phrase *push an argument*. In a related study, Gibbs et al. (2006) studied the ability of a group of participants to form mental images of metaphorical actions that are physically implausible in reality, such as *grasping ideas* and *digesting ideas*. Their results suggested that certain aspects of bodily engagement (watching or imitating) can enhance participants’ abilities to visualize or form mental images for metaphorical actions impossible in reality. Galinsky and Glucksberg (2000) conducted several experiments to examine the priming impact of contextual meanings of prime words. For example, participants encountered the word *fire* in one of the two different figurative senses (playing with fire, being on fire) or a literal sense (playing by the fire). They found that the priming effects of the word *fire* varied depending on the context in which it was presented. Based on these findings, they concluded that the priming effects of words are highly contingent upon their context-appropriate meanings.

Despite the insights gained from these reviewed studies, none investigated the impact of literal and gestural representation of metaphor schemas on the comprehension of subsequent metaphors. To address this gap, we sought to investigate how sensibility judgments on a metaphor are influenced when the metaphor is preceded by a congruent or opposite literal-prime sentence (Stage 1 of our experiment) or a congruent or opposite gesture-prime schema (Stage 2 of our experiment). We intended to answer the following questions:

1. Does a congruent or opposite literal-prime sentence influence the process of metaphor comprehension?
2. Does a congruent or opposite gesture-prime schema affect the process of metaphor comprehension?

To explore these research questions, we collected sensibility judgments and reaction times in both stages of the experiment. In each stage of the experiment, a comparison was made between congruent and opposite conditions. It was hypothesized that congruent literal-prime and congruent gesture-prime conditions would facilitate the process of understanding the subsequent metaphors, as reflected in faster judgment times and higher sensibility ratings.

## 3 Method

### 3.1 Participants

One hundred and sixty-three foreign language learners participated in this study (98 females, 65 males, age = 16–52). All of them were native Persian speakers. They were divided into five groups, Group 1 consisted of 32 participants, Group 2 consisted of 31 participants, Group 3 consisted of 36 participants, Group 4 consisted of 33 participants, and Group 5 consisted of 31. Group 1 (congruent literal-prime conditions) and Group 2 (opposite literal-prime conditions) participated in the first stage of the experiment. Group 3 (no-prime conditions) was the baseline group. Group 4 and 5 participated in the second stage of the experiment. Group 3 served as the baseline group for both stages of our experiment. All participants in the study participated voluntarily and gave their written informed consent. The study was conducted in accordance with the Declaration of Helsinki (World Medical Association, 2013).

### 3.2 Procedure

Group 1 (congruent literal-prime conditions): Participants made sensibility judgments on 15 metaphors in congruent literal-prime conditions. For each item, a metaphorical statement was preceded by a five-second video showing a literal sentence containing a congruent schema. For example, in one of the items, the metaphor *these days, billboards are growing warts on the skins of cities* was preceded by the literal-prime sentence *there was a protrusion on the surface of the road*. These two sentences share the schema *X goes up on the surface of Y*, although the first is a literal schema and the second is a metaphor schema. For each item, after observing the literal-prime sentence for 5 s, participants had judge the sensibility of the subsequent metaphor that appeared on the computer screen. Each metaphor was displayed on the screen for a period of 7 s. The participants had to make a sensibility judgment on the metaphor by pressing the button M or Z. Key allocations were counterbalanced between participants. Subsequently, the next item was shown on the screen. Sensibility judgments and the corresponding reaction times for each item were recorded.

Group 2 (Opposite literal-prime conditions): Participants made sensibility judgments on 15 metaphors in opposite literal-prime conditions. Identical metaphors employed for Group 1 were used for Group 2. For each item, a metaphorical statement was preceded by a five-second video showing an opposite literal-prime sentence that contained an opposite schema. For example, in one of the items, the metaphor *these days, billboards are growing warts on the skins of cities* was preceded by the opposite literal-prime sentence *there was a hole on the surface of the road*. These two sentences had opposite schemas, one had the schema *X goes up on the surface of Y* and the other one had the schema *X goes down on the surface of Y*. For each item, after seeing the opposite literal-prime sentence for a period of 5 s, participants had to make a judgment on the sensibility of the subsequent metaphor that appeared on the screen of the computer. Each metaphor was displayed on the screen for a period of 7 s. The participants had to make a sensibility judgment on the metaphor by pressing button M or Z similar to the Group 1 process. Key allocations were counterbalanced between participants. Sensibility judgments and the corresponding reaction times for each item were recorded.

Group 3 (Baseline group, No-prime conditions): Participants made sensibility judgments on 15 metaphors in no-prime conditions. Each metaphor was shown on the screen for a period of 7 s. Sensibility judgments and times of making sensibility judgments were recorded for each item. The same metaphors used for Group 1 and Group 2 were also used for Group 3.

Group 4 (Congruent gesture-prime conditions): Participants made sensibility judgments on 15 metaphors in congruent gesture-prime conditions. The same metaphors used in Stage 1 of our experiment 1 were also used in Stage 2. In each item, a metaphorical statement was preceded by a five-second video displaying the gestural representation of the metaphor. For example, the metaphor *a rumor is a virus* was preceded by a gesture that showed the spread of something in the three-dimensional space. This was the gestural representation of the metaphor schema *X spreads in the space*. For each item, after the observation of the gestural representation of each metaphor schema for a period of 5 s, participants judged the sensibility of the metaphor that appeared on their computer screens. The length of the display for each metaphor was 7 s. Sensibility judgments and reaction times of making sensibility judgments were recorded for each item. The participants judged the sensibility of the metaphor by pressing button M or button Z. Key allocations were counterbalanced between participants. Sensibility judgments and times of making sensibility judgments were recorded for each item.

Group 5 (Opposite gesture-prime conditions): Participants had to make sensibility judgments on 15 metaphors in opposite gesture-prime conditions. Identical metaphors used for previous were also employed for Group 5. For each item, a metaphorical statement was preceded by a five-second video that showed the opposite schema of the metaphor. For example, the metaphor *a rumor is a virus* was preceded by a gesture that represented the reversed action of spreading. Take another example, the metaphor *a project development is a forward movement* was preceded by a gesture that showed a backward movement (reversed version of a gesture showing a forward movement). For each item, after observing the opposite gesture-prime of the subsequent metaphor for a period of 5 s, participants had to make a judgment on the sensibility of the metaphor that appeared on the screen of the computer. Each metaphor was shown on the screen for a period of 7 s. Sensibility judgments and reaction times of making sensibility judgments were recorded for each item. The participants had to make a sensibility judgment on the metaphor by pressing button M or button Z. Key allocations were counterbalanced between participants.

### 3.3 Design

In one part of the study, sensibilities of metaphors for various groups were compared (congruent literal-prime group, opposite literal-prime group, no-prime group, congruent gesture-prime group, and opposite gesture-prime group). In another part of the study, times of making sensibility judgments in various conditions were compared (congruent literal-prime conditions, opposite literal-prime conditions, no-prime conditions, congruent gesture-prime conditions, and opposite gesture-prime conditions). In other words, reactions of different groups in different conditions were compared with one another. These reactions included sensibility (sensible or not sensibile) and time of providing sensibility judgment. Therefore, our study had a between-subject design.

### 3.4 Data analysis

Two ANOVA tests were used. The first one was used to make a comparison among sensibility of metaphors in Stage 1 (Group 1, Group 2, and Group 3 as the baseline) and Stage 2 (Group 3 as the baseline, Group 4, and Group 5). The aim was to make a comparison among degrees of sensibility of metaphors in congruent literal-prime conditions, opposite literal-prime conditions, no-prime conditions, congruent gesture-prime conditions, and opposite gesture-prime conditions. The second ANOVA test was used to make a comparison among latencies of making sensibility judgments in Stage 1 and Stage 2. The data were analyzed by Rstudio software.

## 4 Results

**Figure 1:**
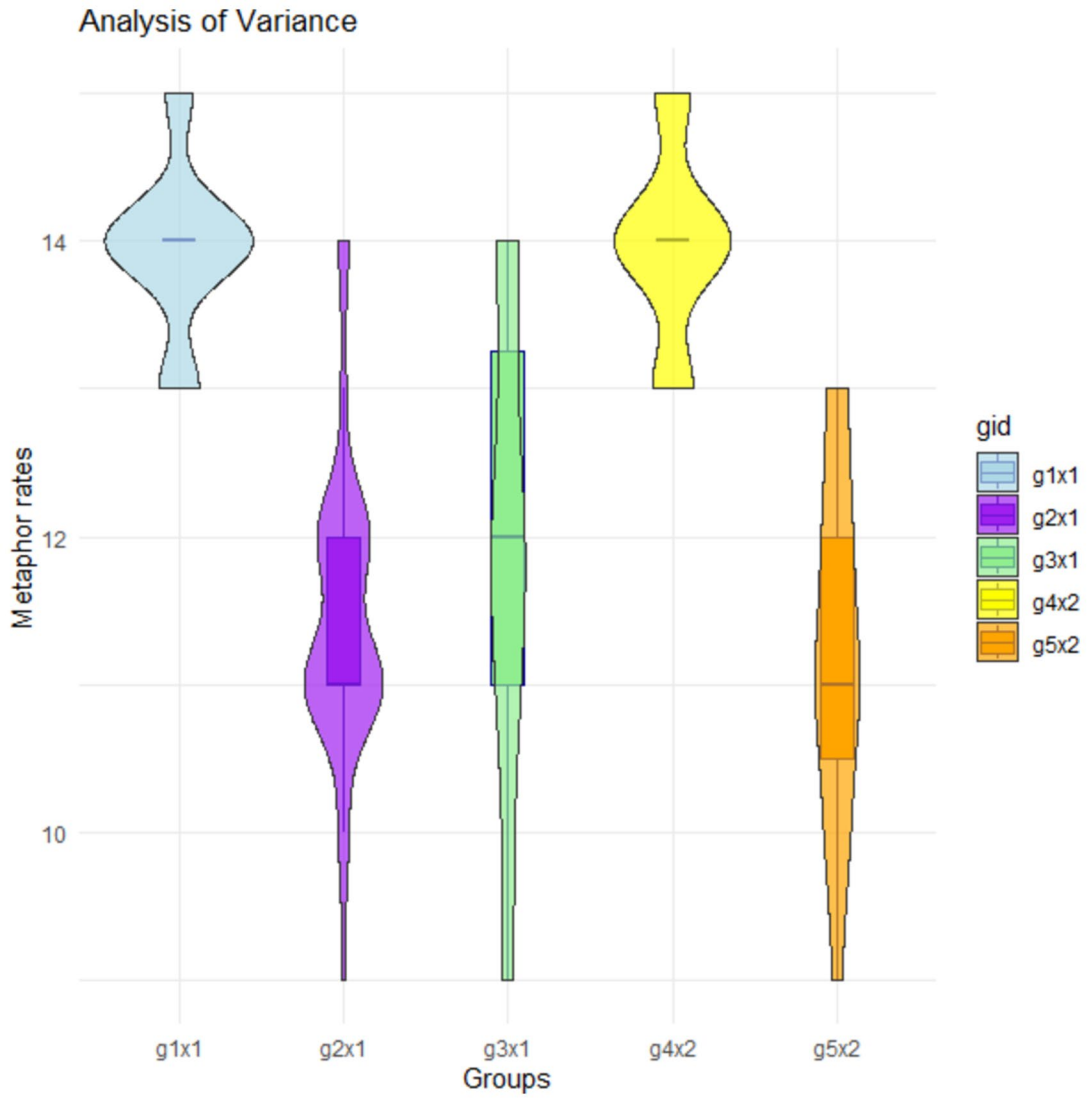
Results of comparing sensibility judgments across the five groups (g1 × 1 = group 1, stage 1; g2 × 1 = group 2, stage 1; g3 × 1 = group 3, stage 1; g4 × 2 = group 4, stage 2; g5 × 2 = group 5, stage 2).

Results of the initial ANOVA test revealed a highly significant F-statistic of 50.603 (*p*-value <0.001), indicating substantial inter-group variance. The mean square for the between-groups effect was 58.511. In Stage 1 of the experiment, participants of Group 1 exhibited the highest degree of metaphor sensibility, whereas participants of Group 2 gave the lowest degree of sensibility. Likewise, in Stage 2 of the experiment, among Group 3, Group 4, and Group 5, participants of Group 4 showed the highest degree of sensibility to metaphors, while Group 5 participants showed the lowest. An Eta value of 0.749 indicated a robust positive relationship between degree of metaphor sensibility and group classification. Similarly, the Eta-square value of 0.562 suggested that approximately 56.2% of the variance in the degrees of metaphor sensibility could be attributed to group differences, as shown in Figure 1.

**Figure 2:**
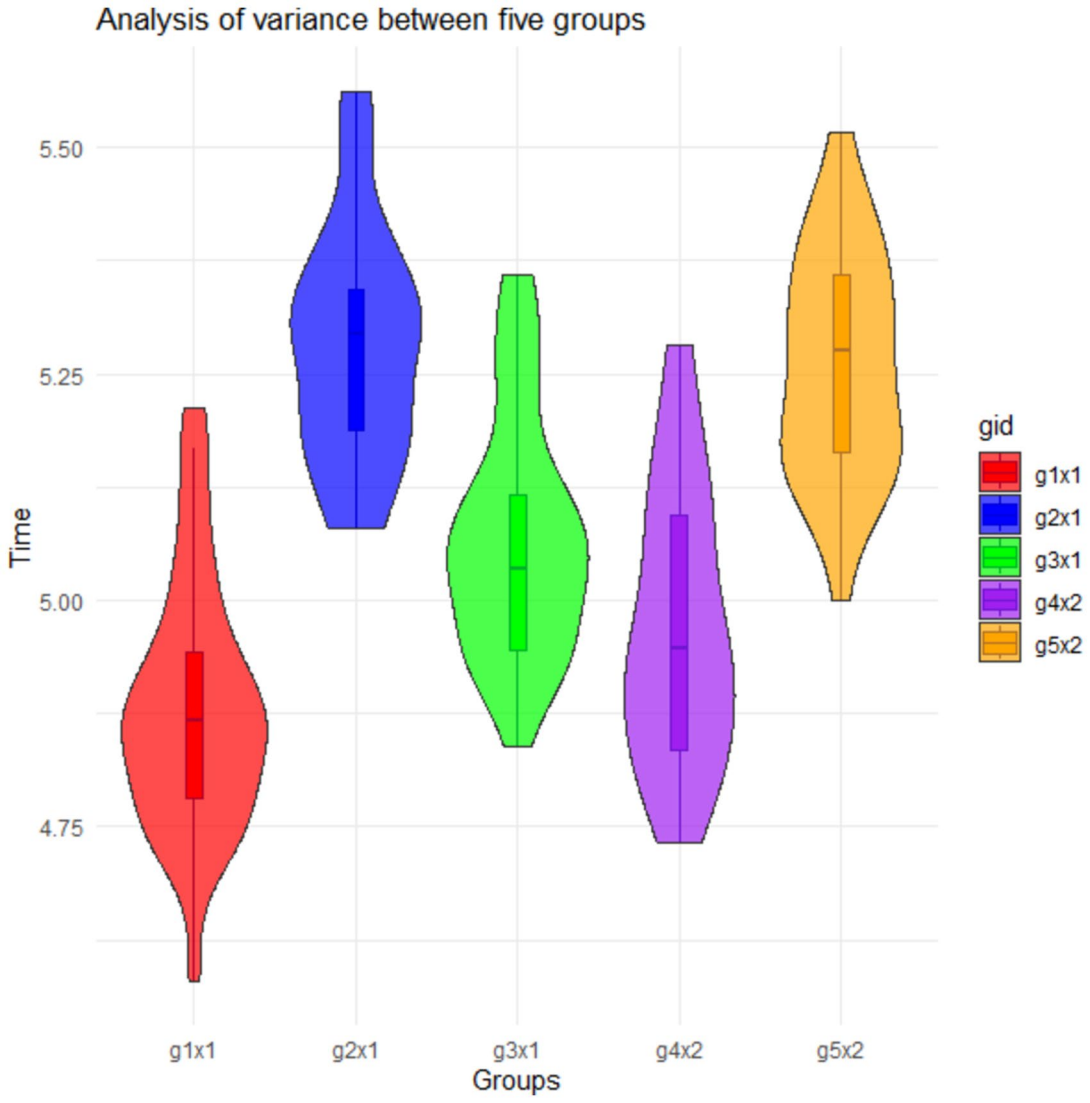
Results of comparing sensibility judgment times across the five groups.

Subsequent ANOVA test results also showed significant variations in sensibility judgment times across the five groups, evidenced by an F-statistic of 53.789 (*p*-value <0.001). This suggests that these variations were not due to random chance, supported by a between-groups mean square of 0.976 and a within-groups mean square of 0.018. These results indicate notable differences in sensibility judgments across the five groups. In Stage 1 of the experiment, among Group 1, Group 2, and Group 3, participants of Group 1 made the quickest judgments (shortest latencies), while participants of Group 2 made the slowest judgments (longest latencies). In Stage 2 of the experiment, among Group 3, Group 4, and Group 5, participants of Group 4 made the quickest judgments, while participants of group 5 made the slowest judgments. The Eta value of 0.759 indicated a strong positive relationship between times of making sensibility judgments and the groups. An Eta-squared value of 0.577 suggested that approximately 57.7% of the variability in sensibility judgment times could be explained by group differences, as shown in Figure 2.

## 5 Discussion

### 5.1 Congruent literal-prime conditions vs. opposite literal-prime conditions

Results obtained from Stage 1 of our experiment showed that response times were significantly shorter in congruent literal-prime conditions, where each metaphor and its preceding literal statement shared the same schema. For instance, the metaphor *the criminal fell into the net that awaited him* was preceded by the literal statement *the bird was captured by hunters*. Conversely, in opposite literal-prime conditions, each metaphor and its preceding literal statement had opposite schemas. An example is the metaphor *the criminal fell into the net that awaited him* was preceded by the literal statement *the bird was released by its owner*. A pertinent question is how a literal prime can facilitate the processing of the subsequent metaphor. One explanation that can be offered for the priming effect of congruent literal prime is that congruent literal-prime sentence activates a key component of the meaning of the subsequent metaphor, thus easing the interpretation process. For example, a significant part of the meaning of the metaphor *the criminal fell into the net that awaited him* is the notion of someone being captured or held by a powerful force. This is the schema of this metaphor and embodies an essential part of the meaning of this metaphor. If this schematic part of the meaning is activated in the mind of a comprehender by a preceding prime such as a congruent literal prime, the metaphor’s comprehension can be facilitated.

From the perspective of the strong versions of embodiment theories (Gallese and Lakoff, 2005), when the comprehender processes the congruent literal-prime sentence, s/he embodies the schema of capturing something by a strong force. This process likely activates sensorimotor networks associated with the physical enactment of such capturing actions corresponding to the exertion of a strong force. This schema and the sensorimotor networks that represent it along with the actions that are involved in it remain active for a limited duration. The persistence of this active state of this schema and the sensorimotor networks that represent this schema and the action that are involved in it has the potential to facilitate the understanding of the subsequent statement, provided that they engage the same underlying schema. Importantly, this facilitation can occur even if the subsequent statement is a metaphorical statement that contains the same schema. This view is consistent with a proposal suggested by Gibbs et al. (2006), according to which people can create coherent mental images of metaphorical actions even when those metaphorical actions are not physically possible in reality. In fact, this suggests that people possess the cognitive capacity to create mental images of both possible and impossible actions. The activation of such mental images can facilitate the process of understanding literal and metaphorical sentences.

A critical point to note here is that, in Stage 1 of the experiment, the actions associated with the schema of each literal-prime sentence were physically possible in the real world. In contrast, the actions associated with the schema of each metaphor were not. However, it seems that the physical possibility or impossibility of the action associated with each schema appears to be inconsequential in the processing of these sentences. The key factor is that the comprehender’s ability to create a schema (whether it represents a possible or impossible situation) and to interpret that metaphor on the basis of this schema. In fact, the important part of information is schema-dependent, suggesting that the feasibility of the action associated with the schema in the real world is inconsequential.

The findings from Stage 1 of the experiment suggest that metaphor schema can play an integral role in the meaning of the metaphor, and it is in some kind of interaction with other semantic aspects of the metaphor. Once a metaphor schema is activated, the activation of other components of the metaphor’s meaning is facilitated. On the other hand, when a schema that is antithetical to the schema of a metaphor is activated immediately before metaphor processing, its comprehension may be delayed. This is supported by the results obtained from Stage 2 of the experiment. When the schema activated by a literal prime conflicts with the schema of the subsequent metaphor, the comprehender requires some time to suppress that schema. In other words, since the schema that is activated by the literal prime is not congruent with the intended meaning of the subsequent metaphor, it interferes with the process of understanding and delays the process of making a sensibility judgment on the metaphor. For example, the schema invoked by the literal sentence *he picked up an object that was on the corner of the room* opposes the schema of the metaphorical sentence *you need to put aside the habit of being late at work*. Processing the literal sentence activates the schema and the action of physically picking up an object in the comprehender’s mind. When the literal sentence is presented before the metaphorical sentence, the schema of the literal sentence must be suppressed as it conflicts with the intended schema of the subsequent metaphor. Suppression of semantic features is widely acknowledged as a crucial part of metaphor comprehension (e.g., Banaruee et al., 2017, 2019a, 2019b; Khatin-Zadeh et al., 2019).

### 5.2 Congruent gesture-prime conditions vs. opposite gesture-prime conditions

As previously mentioned, the results of our experiment for group 3, group 4, and group 5 showed that participants were faster in making sensibility judgments in congruent gesture-prime conditions than opposite gesture-prime and no-prime conditions. Additionally, metaphors exhibited a higher degree of sensibility in congruent gesture-prime conditions. In congruent gesture-prime conditions, each metaphor was preceded by a gestural representation of its underlying schema. These findings align with the results obtained for groups 1, 2, and 3, reinforcing the idea that that the activation of metaphor schema with its gestural representation can facilitate the process of understanding the subsequent metaphor. Therefore, the gestural representation of a metaphor schema appears to convey an significant portion of the metaphor’s meaning.

In an analogous way that congruent literal-prime sentence can facilitate the process of activating metaphorically-relevant information, the congruent gesture-prime can activate the core part of metaphorical meaning, directing the comprehender’s attention toward metaphorically-relevant information. For example, when the metaphor *the project is moving forward and will reach its destination by the end of the year* is preceded by a gesture that depicts a forward movement, the schema of this metaphor is activated in the mind of the comprehender.

This, in turn, facilitates the process of activating metaphorically-relevant information and accelerates the process of metaphor comprehension. Conversely, when this metaphor is preceded by a gesture illustrating a backward movement (opposite gesture-prime conditions), the gesture may activate metaphorically-irrelevant information. The activation of such metaphorically-irrelevant or contradictory information may interfere with the process of understanding the subsequent metaphor, which may explain why participants were slower in making sensibility judgments in opposite gesture-prime conditions. Nevertheless, it is important to note that the participants in this study where limited to one cultural and linguistic context. Consequently, further cross-cultural research may be crucial to test the generalizability of these findings, since metaphors can be to some extent culture-specific (see Banaruee et al., 2024; Farsani et al., 2021, 2022; Khatin-Zadeh et al., 2023c; Rosa et al., 2020).

Gestures present a visual description of the message, aiding the listener in focusing on the most relevant aspects of the information (Rosa and Farsani, 2021; Farsani and Villa-Ochoa, 2022). This is corroborated by numerous studies that have demonstrated the pivotal role of gestures and other visual aids in the processing of abstract concepts (e.g., Bobek and Tversky, 2016; Hegarty et al., 1990; Hegarty and Just, 1993). The contribution of gestures to the understanding of metaphorical statements becomes particularly salient in instances where key elements of metaphorical meaning can be visually depicted. For example, the central aspect of the meaning of the metaphor *the rumor became viral* can be shown by a gesture illustrating the spread of something in an area. When such critical information is shown by a gesture, the interpretation of this metaphor can be facilitated. Therefore, it can be suggested that the degree of contribution of gestures to metaphor comprehension is dependent on what part of information can be shown by gestures. If the key metaphorical component of meaning can be effectively conveyed through a gesture, that gesture can significantly enhance the speed and accuracy of processing that metaphor.

### 5.3 Semantic features defining the metaphorical meaning

This section interprets the study’s results from the perspective of a proposal put forth by Khatin-Zadeh and Vahdat (2015). According to this proposal, metaphorical meaning of a given term is usually defined by a single semantic feature or a very small set of closely related semantic features. For instance, in the metaphor *rumors are viruses*, the metaphorical meaning of *virus* is defined by the single semantic feature of “rapid spread.” During the processing of this metaphor, this key semantic feature becomes salient, while other semantic features, which are metaphorically irrelevant, are suppressed (see Banaruee et al., 2017; Khatin-Zadeh and Vahdat, 2015). From the perspective, the defining feature of a metaphorical meaning is closely tied to its underlying metaphor schema. In the case of the metaphor *rumors are viruses*, the schema of this metaphor shows the spread of small objects across an area in the space, while the semantic feature of “rapid spread” is the defining feature of the metaphorical meaning of *virus*. This semantic feature is intimately connected to the schema of the metaphor *rumors are viruses*.

Consider another example: the schema of the metaphor *the criminal fell into the net that awaited him* represents the capture of an individual. The feature of “capture” is the central semantic feature that defines the metaphorical meaning of the term *net*. As with the previous example, there is a close relationship between the key semantic feature that defines the metaphorical meaning and the schema of the metaphor. According to Khatin-Zadeh and Vahdat (2015), metaphorical meanings do not describe the concrete details of a concept or an event; rather, they provide a generalization, focusing on one aspect of a concept or an event. When it is said *the criminal fell into the net*, the key conveyed information is that the criminal was captured. The metaphorical statement does not describe the concrete details of the event. The key metaphorical meaning is expressed by a single semantic feature or a single metaphor schema (the schema of capturing an individual or an object). Metaphorical statements are general due to the fact that they describe a concept or an event on the basis of a single semantic feature or a single schema leaving out specific concrete details. Thus, the process of metaphorization can be understood as metaphorical schematization. We define schematization as a process through which the key or task-relevant information of a concept is maintained, while unnecessary or task-irrelevant information is removed. Schematization is a general cognitive process and can occur in various forms. In our study, schematization occurred in two ways. In each item that was presented to groups 1, 2, and 3, a schema and some literal information were activated during the priming stage. When the comprehender was exposed to the subsequent metaphor, the schema was maintained (since it was congruent with the subsequent metaphor) while the literal irrelevant information was deleted or suppressed. In each item that was presented to groups 3, 4, and 5, a schema was activated by its gestural representation. This took place through gestural schematization.

Gestural schematization is a process through which some elements of a representation are removed, while others are maintained (Goldin-Meadow, 2015; Kita et al., 2017). Gestural schematization primarily maintains spatio-motoric information allowing the individual to focus attention on spatio-motoric information (e.g., Beilock and Goldin-Meadow, 2010; Hostetter and Alibali, 2019). In our experiment, gestures activated key schematized information and facilitated the comprehension of subsequent metaphors. Therefore, metaphoric schematization (metaphorization) and gestural schematization are inherently similar processes: both eliminate contextually irrelevant information and preserve key schematic content. In essence, both metaphorical schematization and gestural schematization are special types of symbolization. While metaphorical schematization operates in the verbal domain, gestural schematization functions in the non-verbal domain.

## 6 Conclusion

Our findings underscore the pivotal role of metaphor schema in the comprehension of metaphors. Activation of metaphor schema, either through literal primes or gestural representations, significantly enhances the ease of metaphor understanding. Conversely, the activation of an opposite schema, whether through literal primes or gestural representations, hinders this process, thereby delaying comprehension. These results robustly support theories of embodied metaphor comprehension. Finally, in our study, we investigated the priming effect of gestures that have a meaningful association with subsequent metaphors. The priming effect of gestures that do not have any association with semantic content of subsequent metaphors (e.g., pragmatic gestures, beat gestures) is a question that can be examined in future studies. Another related question that can be investigated in future studies is the timing of gestures that co-occur with metaphoric sentences. A metaporic gesture conveying metaphoric meaning of a sentence may precede or co-occur with the metaphoric sentence. The possibility of any difference between these two conditions is another question that can be investigated in future studies.

## Data Availability

The raw data supporting the conclusions of this article will be made available by the authors, without undue reservation.
